# Islet delta-cell architecture is remodelled in the human pancreas during type 1 diabetes

**DOI:** 10.1038/s41598-025-04471-w

**Published:** 2025-06-05

**Authors:** Angie Tegehall, Olle Korsgren, Sofie Ingvast, Gajana Gasparyan, Louise Granlund, Marcus Lundberg

**Affiliations:** Department of Immunology, Genetics and Pathology, Uppsala University, Uppsala, Sweden

**Keywords:** Delta cell, Type 1 diabetes, Alpha cell, Hypoglycemia, Type 2 diabetes, Somatostatin, Type 1 diabetes, Type 1 diabetes, Type 2 diabetes, Pathogenesis

## Abstract

Delta cells participate in regulating hormone secretion in adjacent alpha- and beta cells and a general assumption is that cells with a shorter distance to the secreting cell receive a higher concentration of the secretory compounds. Isolated islets obtained from donors with type 1 diabetes have a reduced glucagon secretion during low glucose levels, but adding a somatostatin receptor inhibitor increases the glucagon secretion. Despite this, information regarding the delta-cell architecture during diabetes is sparse. The aim of the current study was to determine intra-islet and extra-islet delta-cell architecture in the pancreas during long-standing type 1 diabetes or type 2 diabetes. Pancreatic tissue from nine donors with long-standing type 1 diabetes, six donors with type 2 diabetes, and 13 donors without diabetes were obtained. Sections co-stained for somatostatin, glucagon, and insulin were manually examined. There was an approximately two-fold higher number of alpha cells directly adherent to delta cells in subjects with type 1 diabetes compared with non-diabetic subjects. The delta cells were more peripherally located within the islets of donors with type 1 diabetes. The density of extra-islet single delta cells in the acinar region was more than three-fold higher in type 1 diabetes compared with non-diabetic subjects. No differences in delta-cell architecture could be determined in type 2 diabetes compared to non-diabetic subjects. In conclusion, the islet delta-cell architecture in human type 1 diabetes is remodelled. The higher number of alpha cells directly adherent to delta cells in type 1 diabetes likely increases the alpha cells’ exposure to somatostatin. This finding may be a link partly explaining the reduced glucagon response to hypoglycemia in type 1 diabetes.

**Supplementary Information:**

The online version contains supplementary material available at 10.1038/s41598-025-04471-w.

## Introduction

The delta cells of the pancreatic islets secrete somatostatin, a powerful paracrine inhibitor of both insulin and glucagon secretion from islet beta- and alpha cells^1,2^. In human type 1 diabetes there is an impaired glucagon response to hypoglycemia^3–6^, and death due to iatrogenic hypoglycemia has been estimated to be as high as 10%^7^. Recent data suggest a clinical benefit of using agents that inhibit somatostatin secretion to prevent hypoglycemia in type 1 diabetes^8^. Similarly, diabetes in animal models is associated with impaired glucagon secretion in response to hypoglycemia; an event corrected by somatostatin receptor antagonists^1,9–11^. Human isolated islets from donors with type 1 diabetes do not secrete glucagon in response to low glucose, but treatment of these with a somatostatin receptor antagonist causes an increase in glucagon secretion^12^. This finding has been postulated to be explained by a lack of electrical beta- to delta-cell coupled inhibition as a consequence of beta-cell loss, leading to hypersecretion of somatostatin, as shown in a non-obese diabetic mouse model of diabetes^12^. However, a general assumption is that cells with a higher proximity to secreting cells receive a higher concentration of the secretory compounds^13^. We hypothesized that an altered delta-cell architecture within the islets could also be a link partly explaining the reduced glucagon response to hypoglycemia in type 1 diabetes.

There is limited knowledge of delta-cell histology in the pancreas during disease, despite them being the third most common endocrine cell type within the islet^14,15^. In 1976, the proportion of delta cells within islets was reported to be increased in two cases with type 1 diabetes^16^, but this has not been confirmed in later studies with larger cohorts of donors^15,17^, where it was also found that the alpha-to-delta cell ratio was unaffected in type 1 diabetes^15^. Similarly, in type 2 diabetes an increased proportion of delta cells was initially reported^18^, but recently a study including a larger cohort of samples stated that delta cell area and proportion were unchanged in autopsy samples from donors with type 2 diabetes^19^. Extra-islet beta-cell density is reduced in type 1 diabetes, and extra-islet alpha-cell density is increased nearly five-fold^20^. However, extra-islet delta-cell density remains unreported, and beyond delta-cell proportion within islets during diabetes, additional information regarding the cell type architecture is even more sparse.

The aim of the current study was to determine the islet and extra-islet delta-cell architecture in pancreatic tissue obtained from subjects with long-standing type 1 diabetes or type 2 diabetes. Special emphasis was put on the direct adherence between delta cells and other endocrine cell types and the intra-islet location of these cells. These investigations can potentially help elucidate the pancreatic paracrine signaling dynamic**s** in diabetes.

## Research design and methods

### Human pancreatic specimen

The research work conducted using human tissue followed the guidelines outlined in the Declaration of Helsinki. Consent for organ donation (for clinical transplantation and for use in research) was obtained via online database (https://www.socialstyrelsen.se/en/apply-and-register/join-the-swedish-nationaldonor-register/) or verbally from the deceased’s next of kin by the attending physician and documented in the medical records of the deceased in accordance with Swedish law and as approved by the Swedish Ethical Review Authority (DNR: 2023-01845-01). All tissue included in the study was procured, stored and analyzed as approved by the Regional Ethics Committee in Uppsala (DNR: 2015/444). Samples from brain-dead organ donors with type 1 diabetes, type 2 diabetes and non-diabetic donors were collected from the biobank. Non-diabetic donors were selected based on no previous history of diabetes, no autoantibodies, and HbA1c < 42 mmol/l. BMI and distribution of sex were similar between the three groups. The non-diabetic donors had an age ranging from 19 to 75 to cover both the ages in the type 1 diabetes and type 2 diabetes donor groups. From each donor, pancreatic tissue samples from the head, body and tail were procured and stained. Due to insufficient tissue quality, some pancreatic regions of some donors were excluded. However, all type 1 diabetes and type 2 diabetes donors included contained at least one sample from another region than the pancreatic polypeptide (PP)-enriched head^21^, which otherwise could have skewed the results. The final study cohort comprised 9 type 1 diabetes, 6 type 2 diabetes, and 13 non-diabetic donors. See Table S1 for specific information of each included donor and SFig1 for an illustration of HbA1c, BMI and age in the donor groups. Age and the pancreatic region were confirmed not to affect the number of delta cells per islet area in the non-diabetic donor group (data not shown). The medical records of the donors were not made available to protect the integrity of the deceased person, and as such the exact duration of disease is unfortunately unknown. However, the donors with type 1 diabetes had been diagnosed for > 1 year and most donors with type 2 diabetes had been diagnosed for several years.

### Immunofluorescent staining of endocrine cells

Tissue samples that had been fixed in formalin and embedded in paraffin were sectioned (6 μm) using a Microm HM355S (Thermo Fisher Scientific, Waltham, Massachusetts). All antigens were unmasked by heat-induced antigen retrieval using pH 6 according to the manufacturer’s instructions (Agilent Technologies, Santa Clara, California, USA). The sections were washed in Tris Buffer Saline solution with 0.05% Tween 20 (TBST), blocked with TBST containing 5% goat serum, and thereafter incubated for one hour at room temperature with primary antibodies against somatostatin (direct-conjugated) and glucagon (direct-conjugated) diluted in the ready-to-use insulin primary antibody; details can be found in Table S2. After rinsing of unbound antibodies, the sections were incubated with the secondary antibody for insulin for 45 min at room temperature (Table S2). Finally, sections were counterstained with Sytox Orange (Life Technologies, Carlsbad, California, USA) and mounted using the S3023 Fluorescence mounting medium (Agilent Technologies).

The sections were scanned with a Zeiss Axioscan 7 slide scanner (Zeiss Group, Oberkochen, Germany). The sections were first prescanned and then random regions (representative image in SFig2) of the section were scanned at 40x magnification. To cover the entire depth of the section, 22 Z-stacks covering a range of 7.98 μm (i.e. approximately 0,36 μm between each stack) were acquired.

### Analysis

The analysis was performed by two independent observers that were blinded to the disease status of the donor, using Zen Lite software (version 3.6). An islet was defined as inter-connected endocrine cells (insulin-, glucagon-, and/or somatostatin-positive cells) with a total area ≥ 500 μm² (corresponding to approximately 25 μm in diameter). A minimum of 10 random islets (with some rare exceptions) were selected per section. The border of the islet was marked, and the area and circularity were determined by the software. Within the islet all somatostatin-positive areas were carefully examined in all layers of the z-stack; only those with a nucleus and a distinctive cell shape were further investigated. The average total number of delta cells examined per group (by the two independent observers) were 1478, 896 and 336.5 in the non-diabetic, type 1 diabetic and type 2 diabetic group respectively. For each confirmed delta cell, the somatostatin-, glucagon- and insulin-positive cells directly adherent to the somatostatin-positive area were counted in all available layers of the z-stack, with the same requirements to be counted as a cell, as the delta cells. Thereafter, for each islet, aggregation of delta cells was further determined by scoring whether the islets contained mainly: (1) single cells without any connection to other delta cells, (2) pairs of two delta cells, or (3) aggregates of ≥ 3 inter-connected delta cells. Additionally, the main location of delta cells within each islet was determined as (1) centrally located, (2) equally scattered throughout the islet, or (3) in the peripheral region of the islet. The average score of aggregation as well as the main location based on all available islets in the donor was determined on a scale of 1 to 3. Finally, the extra-islet delta cell density in the acinar pancreas was determined. In each donor, the islets and acinar areas analyzed were pooled from all available pancreatic regions.

### Statistical analysis

GraphPad Prism software (version 10.1.1) was used for statistical analysis. The non-parametric Kruskal-Wallis test followed by Dunn’s post hoc test was used to compare variables between the different patient groups. Each value compared represented an individual donor. *P* < 0.05 was considered statistically significant. Data are expressed as median.

## Results

### Delta cells had more numerous alpha-cell contacts in Islets from donors with type 1 diabetes

**Fig. 1:**
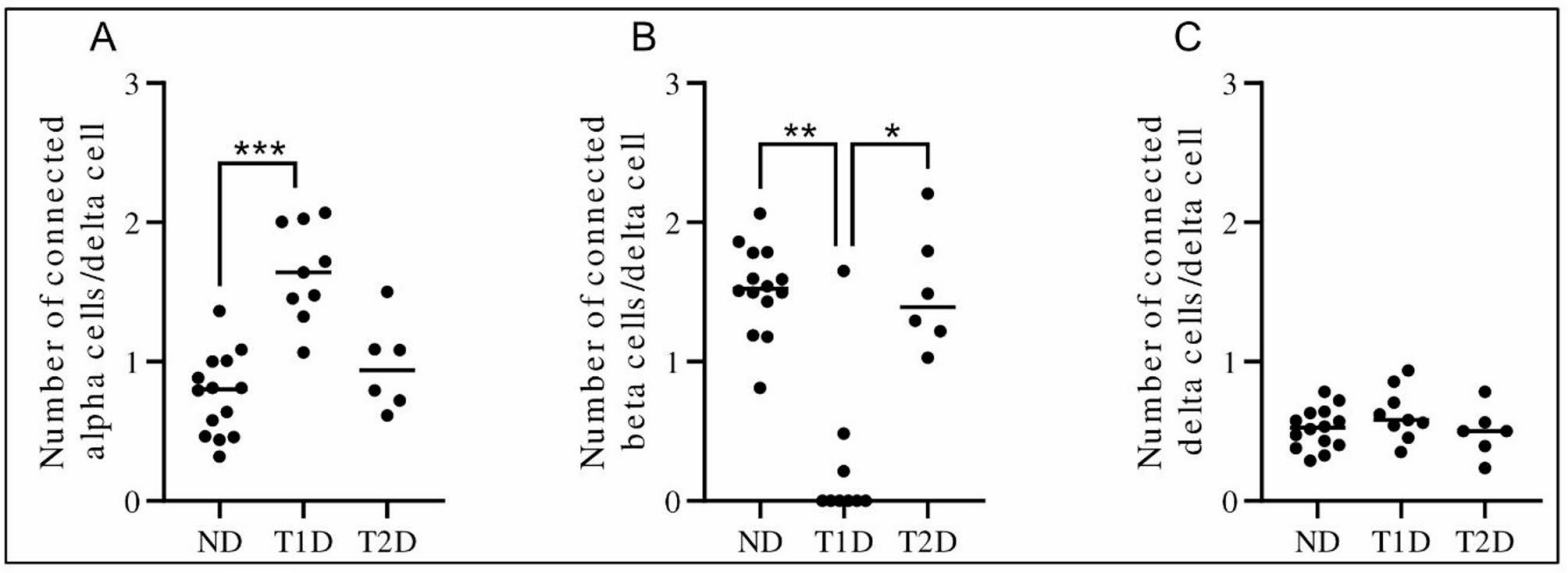
Average number of alpha, beta or delta cells connected to a delta cell. For each confirmed delta cell, the somatostatin-, glucagon- and insulin-positive cells directly adherent to the somatostatin-positive area were counted. The average number of connected cell types was calculated in each donor for all examined cells in the head, body and tail of the pancreas. (A) Number of connected alpha cells per delta cell, (B) number of connected beta cells per delta cell, and (C) number of connected delta cells per delta cell in non-diabetic (ND), type 1 diabetes (T1D) and type 2 diabetes (T2D) pancreases. Each dot represents an individual donor. The bar represents the median value of the group. *, *P* < 0.05, **, *P* < 0.01, ***, *P* < 0.001.

The number of alpha-, beta- and delta cells in direct contact with each delta cell was determined (Fig. 1). The number of alpha cells directly adherent to delta cells was increased in type 1 diabetic (median number of 1.64 connecting alpha cells/delta cell) compared to non-diabetic donors (median number of 0.80 connecting alpha cells/delta cell, *P* = 0.0002), but not compared with type 2 diabetic donors (median number of 0.94 connecting alpha cells/delta cell, *P* = 0.088, Fig. 1A).

The number of beta cells directly adherent to delta cells was reduced in type 1 diabetic (median number of 0.00 connecting beta cells/delta cell) compared to non-diabetic donors (median number of 1.53 connecting beta cells/delta cell, *P* = 0.0015, Fig. 1B), and type 2 diabetic donors (median number of 1.39 connecting beta cells/delta cell, *P* = 0.031 (Fig. 1B). Three of the type 1 diabetic donors had insulin-containing islets in some regions of the section although the majority of islets were insulin-deficient. All other type 1 diabetic cases contained only insulin-deficient islets in the examined sections.

**Fig. 2:**
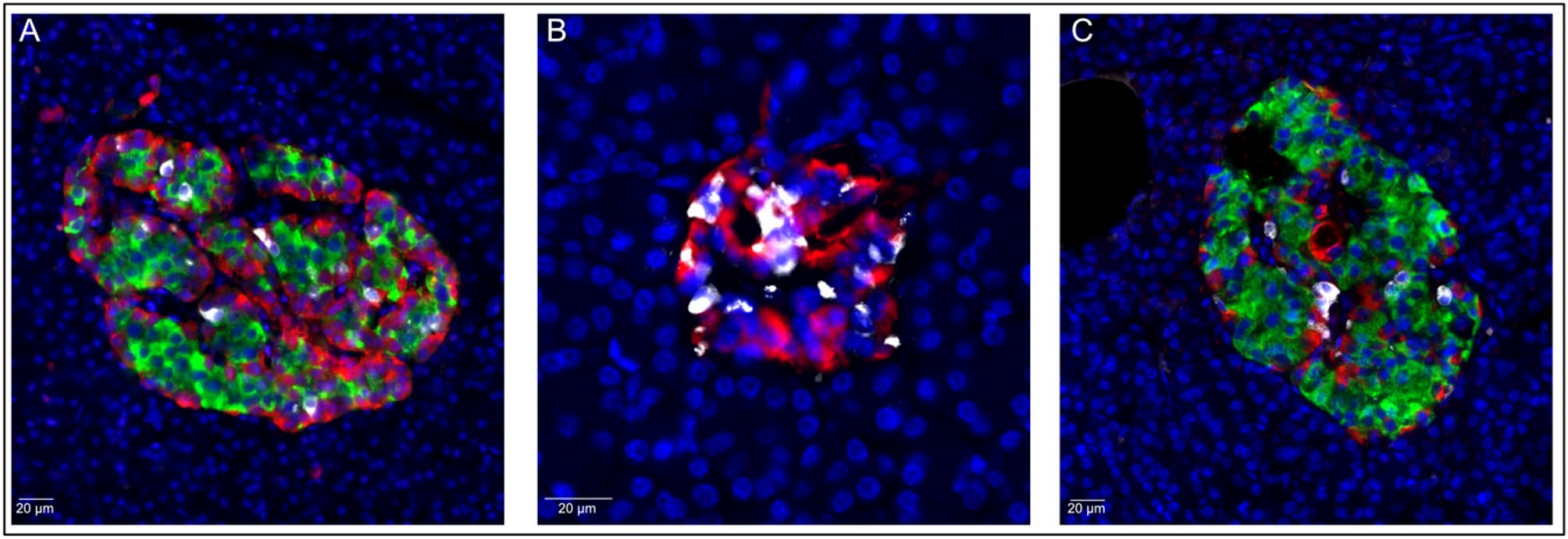
Representative images of islets with immunofluorescent triple-staining of insulin (green), glucagon (red) and somatostatin (white), are shown in a non-diabetic donor (A), a donor with type 1 diabetes (B) and a donor with type 2 diabetes (C). Scale bar: 20 μm.

No difference in the number of delta cells directly adherent to delta cells could be determined between any of the groups (Fig. 1C). Representative images of islets from all donor types are displayed in Fig. 2.

### The delta cells were more peripherally located in Islets from donors with type 1 diabetes

**Fig. 3:**
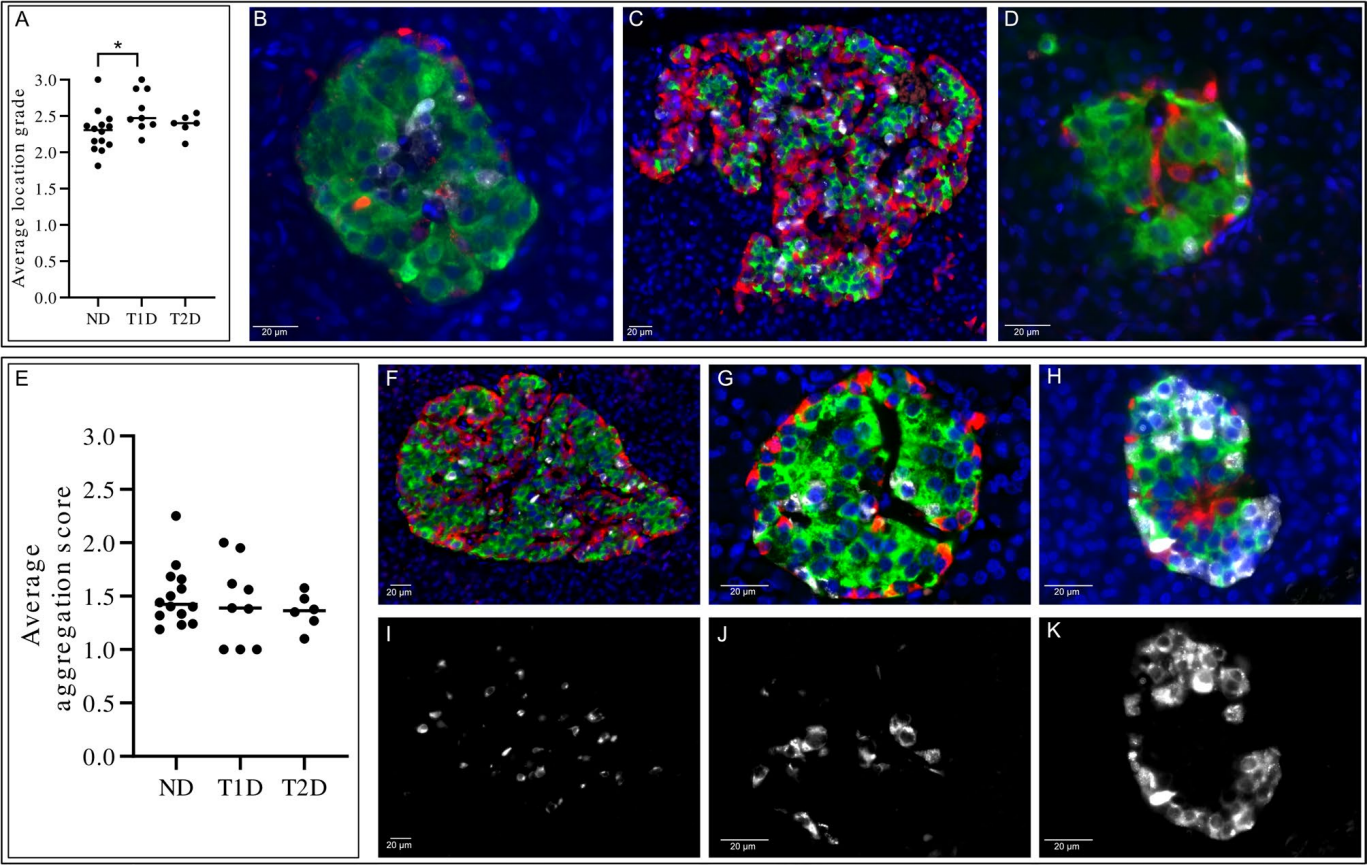
Semi-quantification of delta cell location and aggregation. In each islet it was determined whether delta cells were (1) centrally located, (2) equally distributed throughout the islet or (3) in the peripheral region of the islet. It was also determined whether the islets contained mainly: (1) single delta cells without any connection to other delta cells, (2) pairs of two delta cells, or (3) aggregates of ≥ 3 inter-connected delta cells. (A) The average location grade of all islets in each individual donor was determined in non-diabetic (ND), type 1 diabetes (T1D) and type 2 diabetes (T2D) pancreases. Each dot represents a donor. The bar represents the median value of the group. *, *P* < 0.05. Representative images of islets, based on immunofluorescent triple-staining of insulin (green), glucagon (red) and somatostatin (white) are shown in B-D. (B) Islet showing mainly centrally located delta cells. (C) Islet with delta cells equally distributed throughout the islet. (D) Islet with delta cells mainly located in the peripheral region of the islet. Scale bar: 20 μm. (E) The average aggregation score of all islets in each individual donor was determined in non-diabetic (ND), type 1 diabetes (T1D) and type 2 diabetes (T2D) pancreases. Each dot represents a donor. The bar represents the median value of the group. There were no statistically significant differences between the groups. Representative images of islets, based on immunofluorescent triple-staining of insulin (green), glucagon (red) and somatostatin (white) are shown in F-K. F, I) Islet with mainly single delta cells without any connection to other delta cells. (F) overlay, I) somatostatin. G, J) Islet with mainly pairs of two delta cells. (G) overlay, J) somatostatin. H, K) Islet with mainly aggregates of ≥ 3 inter-connected delta cells. (H) overlay, K) somatostatin. Scale bar: 20 μm.

The main location of delta cells within each islet was determined as (1) centrally located, (2) equally distributed throughout the islet, or (3) in the peripheral region of the islet. The average location of delta cells in each donor was then determined (Fig. 3A-D). Delta cells were on average more prone to a peripheral location within islets from type 1 diabetic donors, compared to islets from non-diabetic donors (*P* = 0.032). No statistical significance was observed between the other groups. Average circularity of islets was reduced in both type 1 diabetic (*P* = 0.0006) and type 2 diabetic (*P* = 0.011) donors compared to non-diabetic donors (SFig3). No statistical significance was observed between donors with type 1 diabetes and type 2 diabetes.

To further examine the interconnections between delta cells, for each islet, aggregation of delta cells was further determined by scoring whether the islets contained mainly: (1) single cells without any connection to other delta cells, (2) pairs of two delta cells, or (3) aggregates of ≥ 3 inter-connected delta cells. The average delta cell state of aggregation per islet was determined in each donor without any differences discovered between the groups (Fig. 3E-K). The average islet area per donor was determined, and the median of these was 6178 µm^2^ in the non-diabetic donor group, 6553 µm^2^ in the type 1 diabetes donor group, and 5500 µm^2^ in the type 2 diabetes donor group (*P* = 0.91). The density of delta cells within the islets was also examined without any differences determined (median non-diabetic donors: 0.64 cells/mm^2^, median type 1 diabetic donors: 0.81 cells/mm^2^, median type 2 diabetic donors: 0.45 cells/mm^2^ SFig4).

### Increased density of extra-islet single delta cells in pancreases from donors with type 1 diabetes

**Fig. 4:**
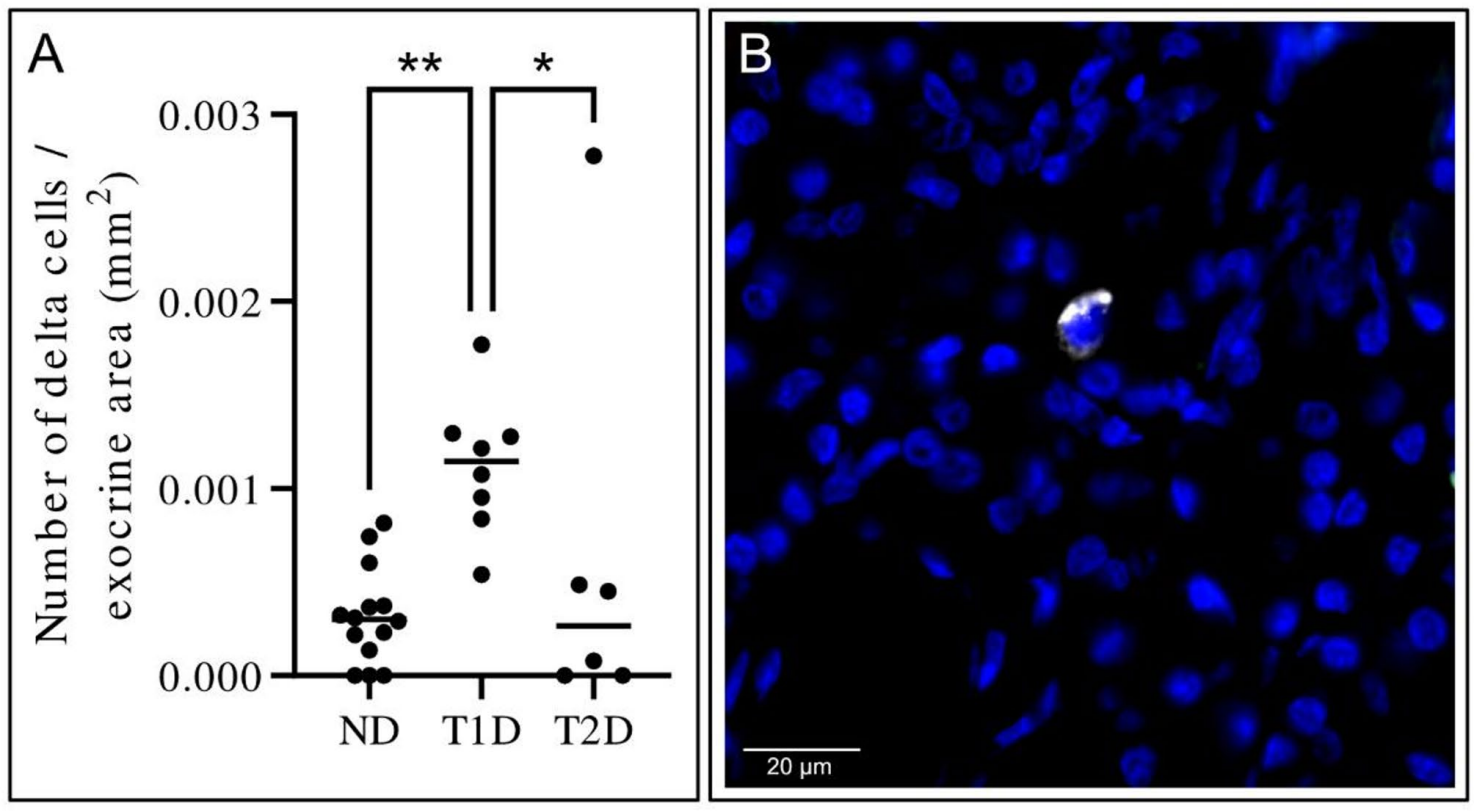
Number of delta cells per exocrine area (mm^2^). (A) Single delta cells in the exocrine region of the pancreas were counted in non-diabetic (ND), type 1 diabetes (T1D) and type 2 diabetes (T2D) donor pancreases. The bar represents the median value of the group. Each dot represents a donor. *, *P* < 0.05, **, *P* < 0.01. (B) Representative image based on immunofluorescent staining of somatostatin (white) and nuclei (blue) illustrating a single delta cell in the exocrine region of the pancreas. Scale bar: 20 μm.

The density of extra-islet single delta cells was determined in the acinar pancreatic region (Fig. 4). Overall, these were sparse in all examined groups but were more numerous in the donors with type 1 diabetes compared to non-diabetic donors (median: 0.0011 delta cells/mm^2^ and 0.0003 delta cells/mm^2^ respectively, *P* = 0.0019) and type 2 diabetic donors (median: 0.00027 delta cells/mm^2^, *P* = 0.032).

## Discussion

Proportions of endocrine cells within islets have been described both in type 1 diabetes and type 2 diabetes previously^15,17–19,22^, with an unaltered alpha-to-delta cell ratio in type 1 diabetes^15^. In the current study, delta cells were examined in well-preserved pancreatic tissue procured from heart-beating organ donors with type 1 diabetes, type 2 diabetes or no previous history of diabetes. We report a median increase of approximately two times more alpha cells in direct adherence to delta cells in type 1 diabetes. Paracrine signaling is dependent on variables such as secretion rate and diffusion rate, however, a general assumption is that cells with a shorter distance to the secreting cell receive a higher concentration of the secretory compounds^13^. Somatostatin inhibitors increase glucagon secretion of isolated islets from donors with type 1 diabetes. Part of this effect is reported to be derived from loss of the beta cells that normally inhibit somatostatin secretion from the delta cells^12^. Based on the results presented herein, we propose that the increased number of alpha cells connected per delta cell in type 1 diabetes could be an additional contributing factor to the impaired glucagon secretion in type 1 diabetes.

Although the islet is composed of only approximately 5% delta cells^23^, filopodia enable contact with distant alpha- and beta-cells^24^. The filopodia are IGF1/VEGF-A responsive and motile^24^. This supports the importance of contacts between the delta cells and other islet cells for the delta cells’ capacity to perform their function. The number of cells connected by delta-cell filopodia could be altered during diabetes which may also influence the paracrine signaling exerted. In pre-diabetic mice there are elongated filopodia and an altered delta-cell morphology^24^. Examination of filopodia requires thick sections which in turn requires non-standardized fixation protocols^24^. With the standard fixation protocol used in the current study, examination of filopodia was unfortunately not possible. We did, however, observe an alteration in delta-cell localization within the islets of Langerhans in subjects with type 1 diabetes, where the delta cells were located more peripherally, instead of being seemingly equally scattered within the islets. The reduced islet circularity could in part explain this finding as it increases the likelihood for an intra-islet cell to be closer to the peripheral region of the islet. Interestingly, the reduction in circularity does not correspond to our previous study^25^. A possible explanation could be that a greater number of controls were included in this study, increasing statistical power.

We have recently reported that the density of extra-islet glucagon-positive cells is increased almost 5-fold in pancreases from donors with type 1 diabetes with a long duration of disease^26^. In the current work we characterized the density of delta cells in the acinar pancreatic tissue. There was an approximately three times higher density of single delta cells in the acinar pancreatic region in type 1 diabetic compared to non-diabetic pancreas. The reason(s) and consequences of this are difficult to speculate on, as these cells to the best of our knowledge have not previously been described in human health or disease. The cells might be proliferating, could have a capacity for further differentiation, they could be part of a neogenetic process originating from ducts^27^, or they are static. However, the low density of these cells, often with only a few cells per section, could argue for a limited function of the cells, and as the presented data only provides a snapshot of the tissue, we cannot determine their role. The reduced pancreas size observed in subjects with type 1 diabetes^28,29^, possibly resulting from loss of acinar but not endocrine cells in the exocrine parenchyma may contribute to the increased density of extra-islet delta- and alpha-cells but seems unlikely to explain the whole phenomenon.

The contact between delta to alpha- or beta-cells has previously been reported to be altered in large islets during type 2 diabetes when each islet is considered a separate sample^19^. In the current study, where a more conservative statistical approach was used by considering every donor a sample, we could not determine any differences in delta-cell architecture in type 2 diabetes compared to non-diabetic subjects. If the loss of beta cells primarily explains the observed differences in type 1 diabetic subjects, the results from type 2 diabetes samples, which showed no apparent indication of beta-cell loss in the examined cohort, would be consistent with this interpretation. Other morphological changes affecting paracrine signaling may exist but remain undetected due to our chosen method. For example, studies have shown that in type 2 diabetes, beta-cell primary cilia are shortened, reducing interactions between beta- and delta-cells^30^.

There are some limitations of the current study. There were no differences in average islet area between the groups, but the current study has not addressed whether alterations in delta-cell architecture were most present in small or large islets. Furthermore, islet area was defined by insulin-, glucagon- and/or somatostatin-positive areas which labels the three most common cell types of the islet. This is expected to give a good approximation of the total islet area, but islets also contain other cell types that were not accounted for in our analysis, which likely affect the islet area to some extent. Z-stacks covering a whole section were analyzed in all available layers, but as the sections were thin, the number of cells may have been underestimated and the position of some cells lost, therefore providing a less well-defined architectural image than a complete 3-D analysis of the islets. Finally, the statistical power was limited due to the sparse access to pancreatic tissue samples from donors with type 1 or type 2 diabetes, but the cohorts included are some of the largest available.

In summary, delta-cell architecture is remodelled in human type 1 diabetes. In type 1 diabetes there are more alpha cells directly adherent to delta cells, likely increasing the alpha cells’ exposure to somatostatin which inhibits glucagon secretion. This may contribute to the reduced glucagon response to hypoglycemia in type 1 diabetes.

## Electronic supplementary material

Below is the link to the electronic supplementary material.

Supplementary Material 1

## Data Availability

All data supporting the findings of this study are available within the paper and its Supplementary Information.
