# Supplementary material for: Islet delta-cell architecture is remodelled in the human pancreas during type 1 diabetes
Source: Sci Rep. 2025 Jun 5;15:19776. doi: 10.1038/s41598-025-04471-w (PMC12141693; doi:10.1038/s41598-025-04471-w)
Supplement: Supplementary file 1 — Supplementary Material 1 [file 41598_2025_4471_MOESM1_ESM.docx]

**Online Supplemental Material**

**Supplementary Table 1.** **Donor characteristics.** Donor type: non-diabetic subjects, subjects with type 1 diabetes or type 2 diabetes, Age, yrs: Age of donor in years at the time of death, Sex: Male or Female gender, BMI: Body mass index, HbA1c in % and mmol/mol, Autoantibodies: autoantibodies present at the time of death, Regions Examined: regions of the pancreas of which analysis were performed on. T1D: subjects with type 1 diabetes. T2D: subjects with type 2 diabetes ND: non-diabetic subjects.

|  |  |  |  | HbA1c | |  |  |
| --- | --- | --- | --- | --- | --- | --- | --- |
| Donor Type | Age | Sex | BMI | % | mmol/mol | Autoantibodies | Regions Examined |
| ND | 62 | M | 38 | 5.8 | 40 | - | Head, tail |
| ND | 21 | M | 20.1 | 5.2 | 33 | - | Head, body, tail |
| ND | 57 | F | 23.9 | 5.3 | 34 | - | Tail |
| ND | 62 | M | 31.4 | 5.7 | 39 | - | Head |
| ND | 54 | F | 30.5 | 5.7 | 39 | - | Body, tail |
| ND | 19 | M | 24 | 5 | 31 | - | Head, body, tail |
| ND | 27 | M | 28.7 | 5.4 | 36 | - | Head, body, tail |
| ND | 36 | M | 24.1 | 5.3 | 34 | - | Head, body, tail |
| ND | 63 | F | 25.2 | 5.4 | 36 | - | Head, body, tail |
| ND | 58 | M | 30.7 | 5.4 | 35 | - | Body |
| ND | 29 | M | 34.3 | 5.4 | 36 | - | Head, body, tail |
| ND | 75 | F | 30.1 | 5.9 | 41 | - | Head, body |
| ND | 46 | M | 24.7 | 5.1 | 32 | - | Body, tail |
| T1D | 16 | M | 21.9 | - | - | - | Head, body, tail |
| T1D | 60 | F | 23.9 | 8.2 | 66 | IA2A, GADA | Head, tail |
| T1D | 68 | M | 30.2 | 8 | 64 | - | Body |
| T1D | 47 | F | 27.6 | 7.4 | 57 | - | Head, tail |
| T1D | 25 | M | 22.8 | 8.9 | 74 | - | Head, body, tail |
| T1D | 24 | M | 27.5 | 8.3 | 67 | IA2A | Head, body, tail |
| T1D | 25 | F | 26.7 | 7.1 | 54 | - | Body |
| T1D | 50 | F | 25.4 | 10.9 | 96 | - | Tail |
| T1D | 70 | F | 18.7 | 5.5 | 37 | - | Head, body |
| T2D | 46 | M | 30.9 | 8.2 | 66 | IA2A | Head, body, tail |
| T2D | 45 | M | 23.4 | 7.3 | 56 | IA2A | Body, tail |
| T2D | 61 | F | 43.3 | 6.6 | 49 | - | Head, body, tail |
| T2D | 59 | M | 26.9 | 6.3 | 45 | - | Head, tail |
| T2D | 63 | F | 37 | 7.9 | 63 | - | Body |
| T2D | 61 | F | 34.1 | 6.3 | 45 | - | Body |

**
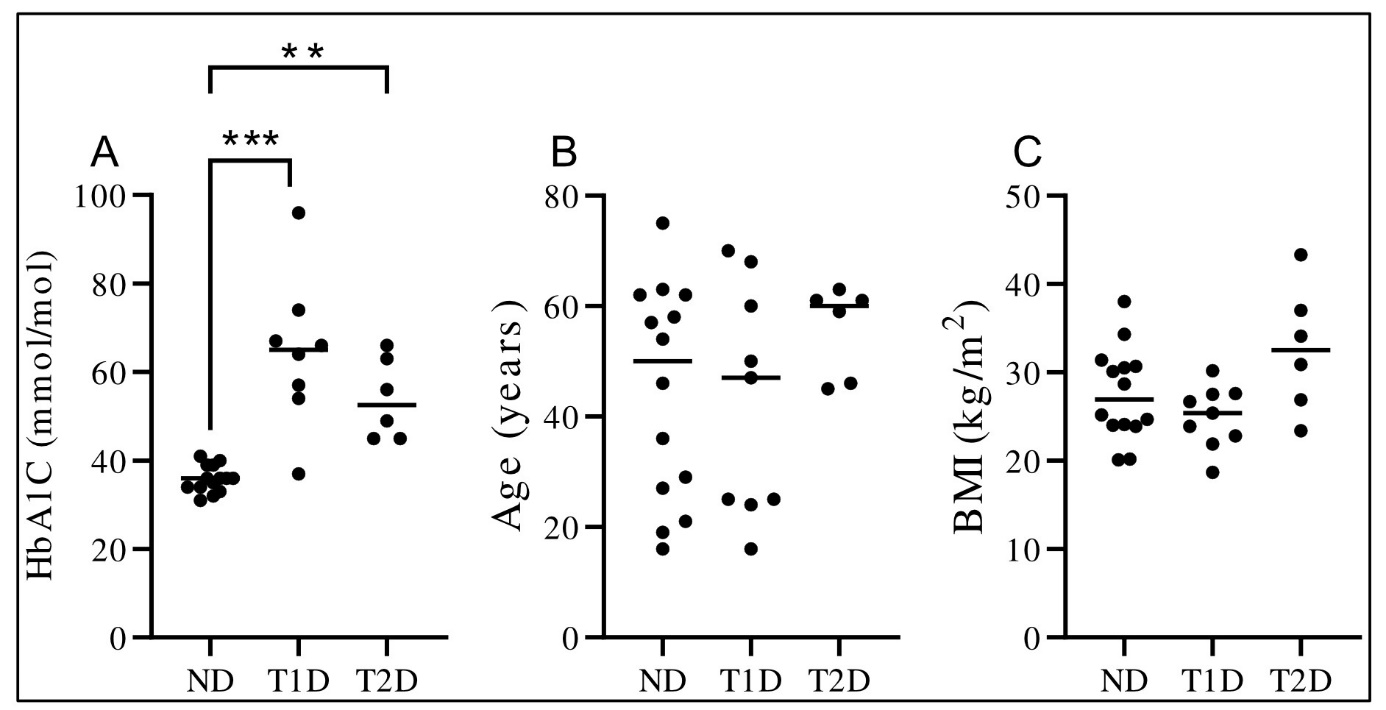
Supplementary figure 1. HbA1c, BMI and age of donors.** The HbA1c (A), age (B), and BMI (C) of the non-diabetic (ND), type 1 diabetes (T1D) and type 2 diabetes (T2D) donors. Each dot represents a donor. The bar represents the median value of the group. **, P<0.01, ***, P<0.001.

**Supplementary Table 2.** Primary antibodies, secondary antibodies and nucleic stains used for immunofluorescence.

| Antibody/Stain | Species | Fluorophore | Concentrations | Catalog # | Company |
| --- | --- | --- | --- | --- | --- |
| Insulin – primary Ab | Polyclonal Guinea Pig | - | Ready-to-use | IR002 | Agilent Technologies |
| Insulin – secondary Ab | Goat-anti-guinea pig | Alexa Fluor 647 | 1:400 | Ab150187 | Abcam |
| Anti-glucagon | Monoclonal Mouse | Brilliant Violet 421 | 1:100 | 565891 | BD Biosciences |
| Anti-somatostatin | Monoclonal Mouse | Alexa Fluor 488 | 1:100 | 566032 | BD Biosciences |
| Sytox Orange |  | Sytox Orange (547/570 nm) | 1:5000 | S11368 | Life Technologies |


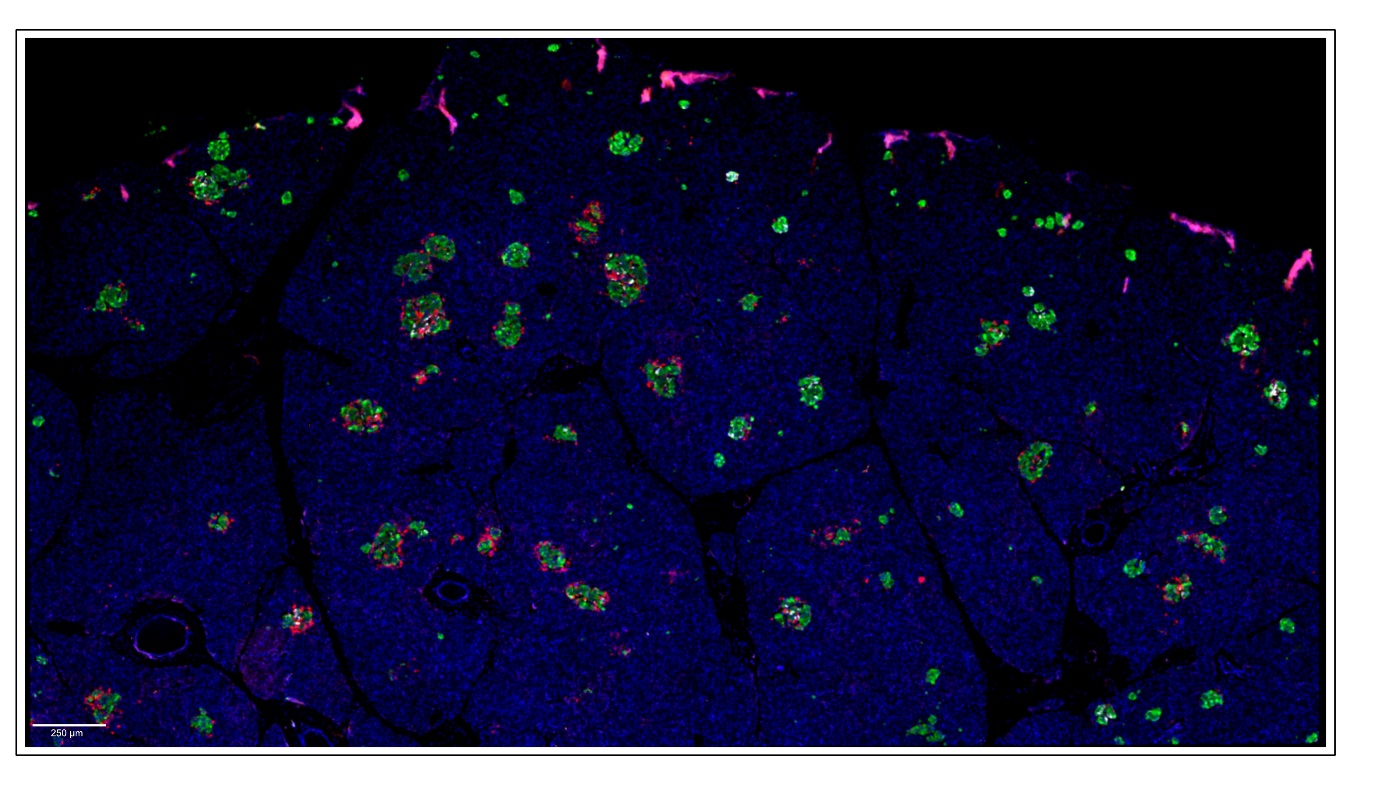
**Supplementary figure 2.** An overview image of a scanned tissue section. Tissue sections were stained using immunofluorescent triple-staining of insulin (green), glucagon (red) and somatostatin (white). Scale bar: 250 µm.

**
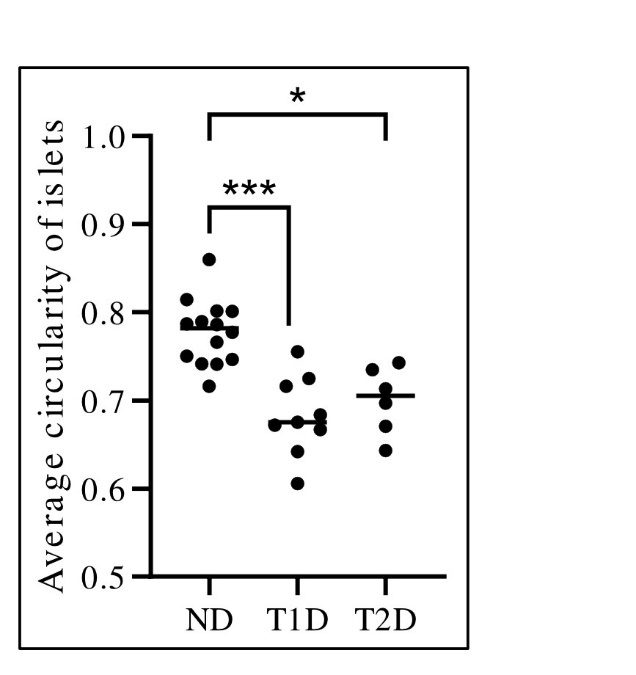
**

**Supplementary figure 3. Circularity of islets.** The circularity of each islet was determined and the average islet circularity was calculated in each donor in non-diabetic (ND), type 1 diabetes (T1D) and type 2 diabetes (T2D) donors. Each dot represents a donor. The bar represents the median value of the group. *, p<0.05, ***, p<0.001.


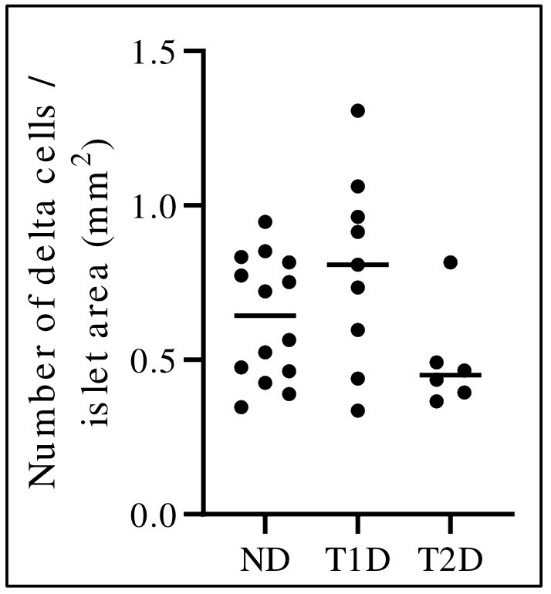


**Supplementary figure 4.** Delta cells per islet area. The number of delta cells per islet area (mm^2^) was determined in each islet and the average was determined in each donor in non-diabetic (ND), type 1 diabetes (T1D) and type 2 diabetes (T2D) pancreases. Each dot represents a donor. The bar represents the median value of the group. There were no statistically significant differences between the groups.
